# Assessment of Knowledge and Awareness of Ultrasonic Bone Surgery Among Periodontology and Oral Surgery Postgraduate Students in Maharashtra

**DOI:** 10.7759/cureus.66812

**Published:** 2024-08-13

**Authors:** Sayali S Dongare, Sameer A Zope, Girish Suragimath, Siddhartha Varma, Apurva V Kale

**Affiliations:** 1 Periodontology, School of Dental Sciences, Krishna Vishwa Vidyapeeth (Deemed to be University), Karad, IND

**Keywords:** ultrasonic bone surgery, piezosurgery, periodontitis, osteoplasty, ostectomy, maxillary sinus lift

## Abstract

Introduction

Ultrasonic bone surgery, or piezosurgery, is a novel technique utilizing ultrasonic vibrations to cut bone while minimizing damage to adjacent soft tissues. This study aims to evaluate the knowledge and awareness of ultrasonic bone surgery among periodontology and oral surgery postgraduate students in Maharashtra, India.

Methods

A cross-sectional, questionnaire-based study was conducted among 180 postgraduate students (84 in periodontology and 96 in oral surgery). A Google Form questionnaire (Google LLC, Mountain View, CA, United States) with 15 close-ended questions was distributed via email. Data were analyzed using SPSS Statistics version 25 (IBM Corp. Released 2017. IBM SPSS Statistics for Windows, Version 25.0. Armonk, NY: IBM Corp.) employing frequency percentages and Pearson Chi-square tests for intergroup comparisons.

Results

Of the participants, 80 (95.3%) of periodontology and 91 (94.7%) of oral surgery postgraduates were aware of ultrasonic bone surgery. Significant differences were found in the knowledge of critical bone damage temperature (p=0.009) and postoperative complications (p=0.037), with periodontology postgraduates showing higher awareness. Both groups recognized ultrasonic bone surgery's precision, safety, and reduced trauma advantages. Overall knowledge and awareness were higher among periodontology postgraduates (n=84, 87.5%) than oral surgery postgraduates (n=96, 78.3%).

Conclusion

Both groups demonstrated a good understanding of ultrasonic bone surgery, with periodontology postgraduates showing notably higher awareness. These findings underscore the importance of integrating ultrasonic bone surgical techniques into dental education to enhance patient care and keep pace with technological advancements. Further studies with larger sample sizes across India are recommended.

## Introduction

Periodontitis is a condition that causes significant damage to the bone that supports the tooth. This damage can lead to bony deformities, which require osseous recontouring to restore the normal bone pattern. In the past, osseous surgery has been performed using either manual or motor-driven instruments [1]. However, both methods have their advantages and disadvantages. A relatively new surgical approach that uses a piezoelectric device has been developed to address these issues. Ultrasonic bone surgery, or piezosurgery, is an advanced technique utilizing ultrasonic vibrations to cut bone tissue precisely while protecting surrounding soft tissues. Traditional manual instruments are difficult to control in cortical bone, and motorized tools often generate excessive heat, causing tissue damage and hindering healing. Ultrasonic bone surgery addresses these issues effectively, making it a revolutionary technology in bone surgery [2]. This is achieved through a piezoelectric transducer, which converts electrical energy into mechanical vibrations. These vibrations are then transferred to a specially designed cutting tip that resonates at a frequency ideal for bone cutting. The main advantages of ultrasonic bone surgery include soft tissue protection, optimal visibility in the surgical field, decreased blood loss, less vibration and noise, and increased comfort for the patient [3]. The main indications in dental surgery are maxillary sinus lift, bone graft harvesting, osteogenic distraction, ridge expansion, endodontic surgery, periodontal surgery [4], inferior alveolar nerve decompression, cyst removal, dental extraction, and impacted tooth removal [5]. It is also indicated in plastic surgeries, spinal surgeries, orthopedic surgeries, otorhinolaryngology [6], skull base procedures, and orbital surgeries. With its ability to provide controlled and precise cutting while minimizing tissue damage, ultrasonic bone surgery is revolutionizing the way surgical interventions are performed [7]. Ultrasonic bone surgery has revolutionized the field of bone surgery by providing a precise, safe, and tissue-friendly method for cutting the bone and making it an appealing choice for modern surgical practices. There are no comparative studies conducted in India regarding knowledge and awareness of applications of this new technology in dentistry among periodontology and oral surgery postgraduate students. Hence, the current study aims to assess the knowledge and awareness of ultrasonic bone surgery among periodontology and oral surgery postgraduate students in Maharashtra.

## Materials and methods

Study design

This cross-sectional questionnaire-based study was carried out among first-, second-, and third-year postgraduate students of periodontology and oral surgery specialties in various dental institutions in Maharashtra. The participant information sheet was provided, and signed electronic consent was obtained from all the participants before enrolling in the study. Postgraduate students who were willing to be part of the study and provided consent were included in the study. Postgraduate students other than those in periodontology and oral surgery were excluded from this study. Anonymous responses were collected to minimize information bias and encourage more honest participant feedback.

Sample size

The sample size of 180 was obtained based on the level of significance (alpha error of 5% and power of 80%) using the formula n=(Z1)2 [P(1-P)]/d2.

Ethical statement

The ethical clearance was obtained from the Ethics Review Committee of Krishna Vishwa Vidyapeeth (ethical clearance protocol number: 201/2022-202, approval date: January 17, 2023) before commencing the study.

Pre-validation and pre-testing of questionnaire

The questionnaire was pre-tested and pre-validated by the Krishna Vishwa Vidyapeeth protocol committee experts. A pilot study among 30 participants was also conducted to assess the reliability and validity of the questionnaire. A specially designed, closed-ended Google Form questionnaire (Google LLC, Mountain View, CA, USA) was created, consisting of 15 questions. Questions were based on two domains regarding knowledge and awareness.

Distribution of questionnaire

The email addresses of postgraduate students were collected by approaching the health institutions in Maharashtra. The digital consent form and questionnaire link were randomly emailed to the periodontology and oral surgery postgraduate students using stratified random sampling. Responses from the participants who filled out both the consent form and questionnaire were considered for statistical analysis.

Statistical analysis

The responses were compiled using Microsoft Excel sheets (Microsoft Corporation, Redmond, WA, USA), and the data was statistically analyzed using SPSS Statistics version 25 (IBM Corp. Released 2017. IBM SPSS Statistics for Windows, Version 25.0. Armonk, NY: IBM Corp.). The basic data was expressed using frequency and percentages. An intergroup comparison between two groups about knowledge and awareness of ultrasonic bone surgery was carried out using the Pearson Chi-square test. A p-value less than 0.05 was considered significant.

## Results

**Figure 1:**
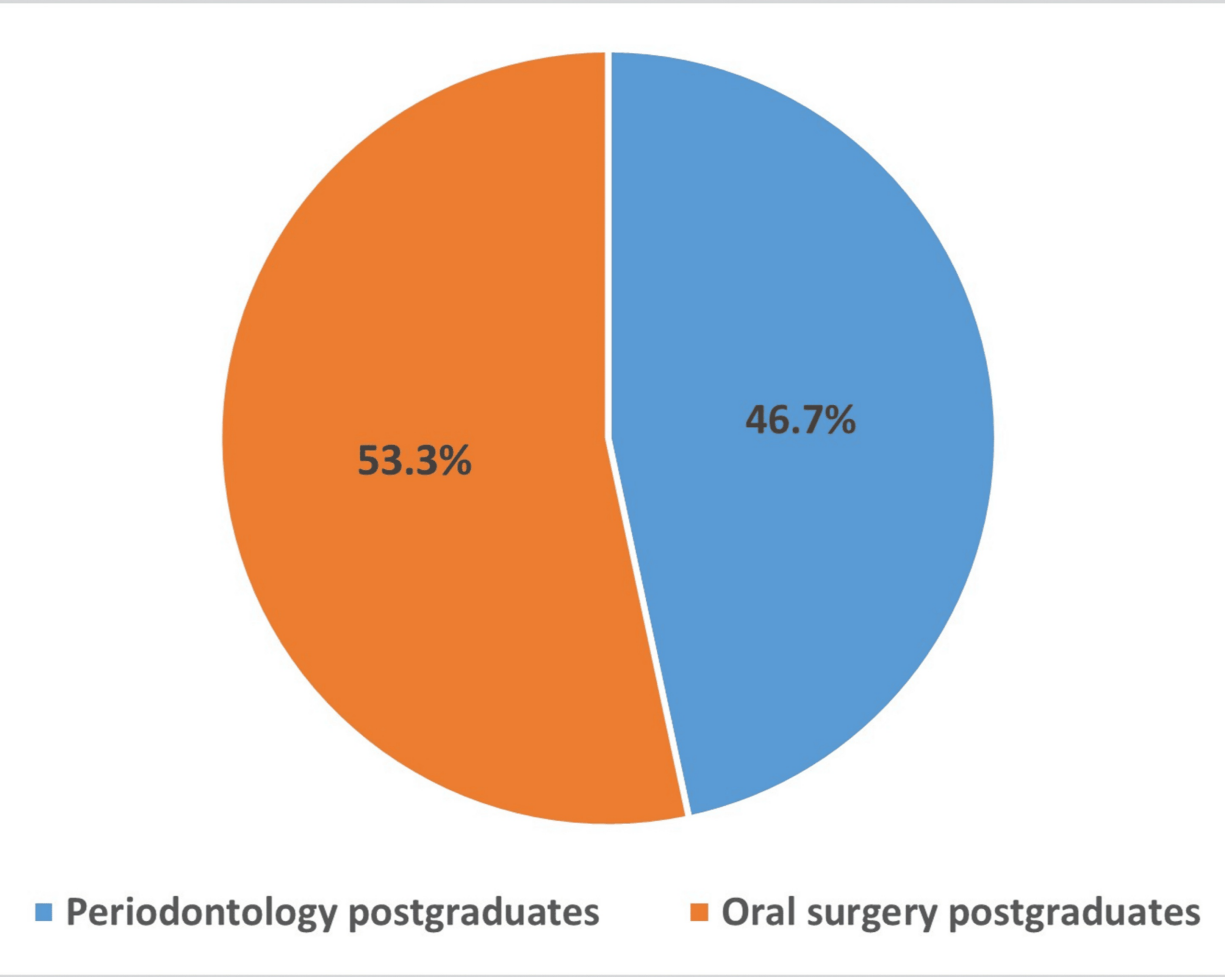
Distribution of postgraduate students

The study aimed to assess the knowledge and awareness of ultrasonic bone surgery among postgraduate students. A total of 180 postgraduate students participated in the study, out of which 84 were periodontology postgraduates and 96 were oral surgery postgraduates (Figure 1).

In the awareness domain (Table 1), 80 (95.3%) of periodontology postgraduates and 91 (94.7%) of oral surgery postgraduates were aware of ultrasonic bone surgery. When asked about the critical temperature for bone damage during bone resection, periodontology postgraduates were more aware than oral surgery postgraduates.

**Table 1:** Questions and responses on awareness of ultrasonic bone surgery * p>0.05 no significant difference

Yes response	Periodontology postgraduates (n=84)	Oral surgery postgraduates (n=96)	Chi-square test	p-value
Aware of ultrasonic bone surgery	80 (95.3%)	91 (94.7%)	1.874	0.392
Aware that 47°C is a critical temperature for bone damage during bone resection	67 (79.7 %)	62 (64.7 %)	9.482	0.009*
Patient compliance is higher while using ultrasonic bone surgery devices	68 (81.2%)	65 (68.1%)	3.605	0.165
Post-operative complications are least in ultrasonic bone surgery than in conventional techniques	76 (90.6%)	78 (75%)	6.616	0.037*
Lack of awareness of ultrasonic bone surgery among the dental fraternity	68 (98.4%)	65 (97.4%)	0.563	0.755
Routine use of ultrasonic bone surgical technique should be given adequate importance in dental education	76 (96.9%)	72 (96.6%)	0.013	0.908

Within the knowledge domain (Table 2), 70 (82.8%) of periodontology postgraduates and 65 (68.1%) of oral surgery postgraduates distinguished that ultrasonic bone surgery could be used for harvesting autogenous bone grafts in bone regenerative therapy, showing statistical significance (p= 0.019). Additionally, both groups agreed that the best frequency range for bone cutting using ultrasonic methods was between 25 and 50 Hz, with 62 (73.4%) of periodontology postgraduates and 70 (72.4%) of oral surgery postgraduates recognizing this range as the most effective. Eighty (95.3%) of periodontology postgraduates accepted that ultrasonic bone surgery devices are expensive when compared with regular cutting instruments.

**Table 2:** Questions and responses on knowledge of ultrasonic bone surgery * p>0.05 no significant difference

Yes response	Periodontology postgraduates (n=84)	Oral surgery postgraduates (n=96)	Chi-square test	p-value
Incorporation of ultrasonic bone surgery in medical disciplines such as plastic and spinal surgery	75 (89.1%)	87 (90.5%)	0.097	0.755
Effective frequency for bone cutting between 25 and 50 Hz	62 (73.4 %)	70 (72.4 %)	4.773	0.092
Ultrasonic bone surgery is more effective than traditional	77 (92.2%)	86 (89.7 %)	0.438	0.803
Used for harvesting autogenous bone grafts in bone regenerative therapy	70 (82.8%)	65 (68.1%)	7.951	0.019*
The cavitation effect associated with ultrasonic bone surgery creates virtually bloodless surgical sites	63 (75%)	(71.6%)	0.508	0.776
Offers the advantage of precise bone cutting over traditional rotary instruments	76 (90.6%)	89 (92.2%)	0.677	0.713
Can patients with pacemakers and cardiopathy be treated with an ultrasonic bone surgery device	4 (4.7%)	6 (6%)	1.034	0.261
More expensive when compared with regular cutting instruments	80 (95.3%)	87 (90.5%)	1.322	0.250
Ultrasonic bone surgery is less traumatic when compared to traditional resective bone surgery techniques	76 (90.6%)	78 (81%)	3.387	0.184

The reported overall knowledge and awareness of ultrasonic bone surgery is 84 (87.50%) for periodontology postgraduate students and 96 (78.30%) for oral surgery postgraduate students.

## Discussion

In the past, when traditional surgical methods were used to treat periodontitis, ostectomy and osteoplasty were the most common surgical treatments. Traditional surgical methods for the treatment of periodontitis had quite a lot of drawbacks, which included time-consuming procedures, overheating of adjacent tissue, multiple instrumentations, a delayed healing process, and difficulty in maintaining a sterile surgical field. So, to overcome these issues, ultrasonic bone surgery was introduced.

Ultrasonic bone surgery is a minimally invasive technique that uses ultrasonic vibrations to cut through bone and soft tissue precisely [8]. Vercellotti et al. developed and patented ultrasonic bone surgery in 1988 [9]. The term "piezo surgery" refers to the use of the piezoelectric effect to generate these ultrasonic vibrations [10]. The tip operates at a specific frequency, usually between 25 kHz and 30 kHz [11]. Ultrasonic vibrations create a cavitation effect in the surrounding fluid, leading to the formation and collapse of microbubbles. Unlike traditional rotary instruments, ultrasonic bone surgery generates minimal heat during the cutting process. This reduced heat production is beneficial for minimizing the risk of thermal damage to surrounding tissues [12]. This effect aids in the cutting process by helping to dislodge and remove debris from the cutting site. One of the advantages of ultrasonic bone surgery is that it allows for selective cutting of mineralized tissues like bone while sparing non-mineralized tissues such as nerves and blood vessels, thereby enhancing the safety and precision of the procedure [13]. Overall, ultrasonic bone surgery offers many benefits, including optimizing precision, minimizing tissue trauma, and ensuring patient safety [14].

The present study was conducted to determine the knowledge and awareness of ultrasonic bone surgery among periodontology and oral surgery postgraduate students in Maharashtra. To the best of our knowledge, our study is the first to be conducted among postgraduate students of periodontology and oral surgery in Maharashtra regarding knowledge and awareness of ultrasonic bone surgery. This study reported that periodontology postgraduates are more knowledgeable and aware of ultrasonic bone surgery and its applications compared to oral surgery postgraduates. In our study, ultrasonic bone surgery was perceived as superior to traditional methods by the majority of postgraduates, with (n=77, 92.2%) of periodontology postgraduates and (n=86, 89.7%) of oral surgery postgraduates indicating their preference for it. Cynthia et al. assessed the knowledge, attitude, and practice of ultrasonic bone surgery among general dentists in the State of Tamil Nadu. They concluded that the majority of dental professionals had good knowledge, attitude, and awareness of ultrasonic bone surgery, and they understood the advantage of using ultrasonic bone surgery over traditional methods [15]. Our study stated that 76 (90.6%) of periodontology postgraduates believed that postoperative complications were less common in ultrasonic bone surgery than in conventional techniques. These findings support the interference given by Arakji et al., who state that ultrasonic bone surgery reduces postoperative pain, trismus, and swelling and enhances the postsurgical quality of the patient’s life [16]. Specifically, 4 (6%) of oral surgery and 6 (4.7%) of periodontology postgraduates indicated yes in their responses, indicating a lack of knowledge of the contraindication of ultrasonic bone surgery in patients with pacemakers. Seventy-five (96.9%) of periodontology and 87 (96.6%) of oral surgery postgraduates agreed that routine use of ultrasonic bone surgery techniques should be given adequate importance in dental education. These results suggest that there is a strong consensus among dental professionals regarding the value of ultrasonic bone surgery techniques in dental education.

These findings highlight the importance of continued education and the integration of ultrasonic bone surgical techniques into dental training programs. The limitation of our study is that it was conducted among postgraduate students of Maharashtra only, and further investigation should be done with a larger sample size for postgraduate students across India to assess knowledge and awareness of ultrasonic bone surgery.

## Conclusions

The data shows that both periodontology and oral surgery postgraduate students possess a good level of knowledge and understanding of ultrasonic bone surgery. However, the study indicates that periodontology postgraduates have a slightly higher level of knowledge and awareness of the technique. Even though there are some differences in their perspectives on various aspects of the method, these findings emphasize the importance of ongoing education and the integration of ultrasonic bone surgical techniques into dental training programs. By doing so, dental professionals can stay up-to-date with the latest advancements and provide high-quality patient care through the application of cutting-edge surgical methods. Therefore, it is crucial to adopt a culture of continuous learning and innovation within dental education to maximize the potential of ultrasonic bone surgery and related technologies in the field of dentistry.
